# Are park availability and satisfaction with neighbourhood parks associated with physical activity and time spent outdoors?

**DOI:** 10.1186/s12889-021-10339-1

**Published:** 2021-02-06

**Authors:** Jenny Veitch, Laura Rodwell, Gavin Abbott, Alison Carver, Elliott Flowers, David Crawford

**Affiliations:** 1 Institute for Physical Activity and Nutrition (IPAN), School of Exercise and Nutrition Sciences, Deakin University, Geelong, Australia; 2 Department for Health Evidence, Section Biostatistics, Radboud Institute for Health Sciences, Radboudumc University Medical Center, Nijmegen, The Netherlands; 3 Mary MacKillop Institute for Health Research, Australian Catholic University, Melbourne, Australia; 4 Institute for Health and Sport, Victoria University, Melbourne, Australia

**Keywords:** Children, Adults, Neighbourhood, Built environment

## Abstract

****Background**:**

There is substantial scope for enhancing population health through increased park visits and active use of parks; however, a better understanding of factors that influence park visitation is needed. This cross-sectional study examined how parent-reported satisfaction and perceived availability of parks were associated with adults’ physical activity and children’s physical activity and time spent outdoors, and whether these associations were mediated by park visitation.

****Methods**:**

Self-reported surveys were completed by adults living within 5 km of two parks located in Melbourne, Australia. Participants reported their satisfaction with neighbourhood park quality, walking duration from home to the nearest park, and park visitation in the past 7 days. Participants with a child aged 2–15 years also answered similar questions in relation to their child. The primary outcome variable for adults was leisure-time physical activity (LTPA) and for children was proxy-reported time spent outside. The secondary outcome for adults was combined transportation and LTPA and for children (5–15 years) was the number of days physical activity recommendations were met in the past 7 days.

**Results:**

Significant positive associations between park availability and park visitation in the past 7 days, and between park visitation and the outcome variables were observed among both adults (*n* = 1085, *M*_*age*_ = 48.9, *SD* 13.4) and children (*n* = 753, *M*_*age*_ = 8.8, *SD* = 3.7). The association between park satisfaction and park visitation was only significant among adults. Park visitation mediated associations between park availability and park satisfaction and the outcome variables among both adults and children.

**Conclusions:**

Improving park availability and users’ satisfaction with parks may increase visitation and consequently increase physical activity and time spent outdoors.

**Supplementary Information:**

The online version contains supplementary material available at 10.1186/s12889-021-10339-1.

## Background

Neighborhood parks can confer multiple health benefits through facilitating physical activity, contact with nature, and social interaction [1, 2], as well as being walkable destinations for residents to engage in recreational physical activity [3]. Despite abundant evidence on the health benefits of parks, they are generally underutilized and visitors often engage in low levels of physical activity during their park visits [4, 5]. A systematic review has reported few differences in park visitation by gender, however, males are more likely to be observed in moderate- to-vigorous intensity physical activity compared to females [6]. Thus, there is substantial scope for enhancing population health through increased visits and active use of parks. However, in order to do so it is necessary to better understand factors that are associated with park visitation (i.e. visiting a park) and physical activity.

Although park availability which includes proximity to home or other places and accessibility have been shown to be positively associated with physical activity among both youth [7–10] and adults [1, 11–13], there are some inconsistencies in the literature [14–19]. A growing body of research indicates that factors other than park availability including factors such as crime and safety [20, 21], park features (e.g. playgrounds, picnic facilities, seating and paths) and quality of amenities and aesthetics (e.g. maintenance, gardens, landscaping) influence park visitation and physical activity [22–24].

We know that people do not always visit their closest park and often travel significant distances from home to reach their preferred park [25, 26]. This may be due to (dis) satisfaction with the features or quality (i.e. unattractive qualities, size, lack of amenities) of the closest park. In an Australian study, the likelihood of adults using public open space increased with increasing levels of access, but the effect was greater after adjusting for distance, attractiveness, and park size. After matching public open space for size and location, 70% of users visited attractive public open spaces [27]. In another study of Australian adults, the presence of a large, high-quality park within walking distance of one’s home was shown to be more important than having other open space closer to home for promoting sufficient amounts of walking for health benefits [28]. Further, among adults aged 57–67 years who walked for recreation, higher park quality was related to greater weekly duration of recreational walking [29].

Despite the importance of park quality, few studies have examined associations between satisfaction with park quality and physical activity among children or youth and this is an existing research gap. A US study of adolescents found that greater perceived park quality (including amenities, maintenance, aesthetics, and safety) was associated with double the odds of park visitation, however, there was no association between park quality and overall objectively measured physical activity [30]. Another study of low-income neighbourhoods in the US found park quality was positively associated with park use and park-based physical activity among children (5–10 years) [31].

It is unclear how park satisfaction (operationalised in our study as self- and parent-reported satisfaction with park quality), park visitation and physical activity are related. Examining whether satisfaction with local parks is associated with park visitation and physical activity in both adults and children will improve our understanding of how parks may facilitate active living. In addition, to our knowledge no studies have considered the pathways through which park satisfaction and perceived availability of parks may operate to influence physical activity; however, park visitation is likely to be on the causal pathway between park satisfaction and physical activity, as well as between park availability and physical activity [24, 32].

**Fig. 1:**
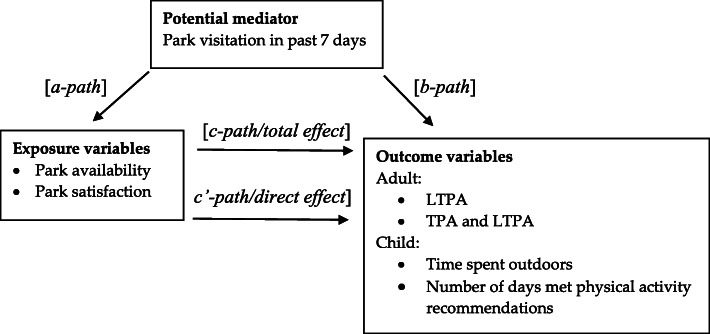
Conceptual model: Park visitation as a mediator of the association between park availability and park satisfaction and physical activity/time spent outdoors

The aims of this study were to examine: 1) satisfaction (with park quality) and perceived availability of parks, and their associations with self-reported physical activity (leisure-time physical activity (LTPA) and transport-related physical activity (TPA)) among adults; 2) parent-reported satisfaction and availability of parks, and their associations with physical activity (time spent outdoors and meeting physical activity recommendations) among children; and 3) whether these associations were mediated by park visitation. It was hypothesised that adults who reported greater satisfaction with the quality of their local parks and greater perceived availability of these parks, and children whose parents reported the above would visit parks more frequently, thus increasing the likelihood of engaging in physical activity and spending time outdoors (see Fig. 1).

## Methods

This study was nested within the Recording and EValuating Activity in a Modified Park (REVAMP) study [33]. Ethics approval was obtained from the University Human Ethics Advisory Group (HEAG-H 46–2012), the Department of Education and Early Childhood Development and the Catholic Education Office Melbourne.

Briefly, the REVAMP study was designed to evaluate the impact of a park modification by using multiple measures to comprehensively assess park visitation and park-based physical activity in two metropolitan parks in Melbourne, Australia: an intervention park and a control park. The intervention park was located 28 km north-west of Melbourne’s central business district (CBD) in a low socio-economic status (SES) area. In 2016, the population for the local city council area where the intervention park was located was 209,523 with 17 persons per hectare, 58% spoke a language other than English at home and 20% had a low household income [34]. The control park was located 22 km east of Melbourne’s CBD in a high SES area. In 2016, the population for the local city council area was 127,573 with 11 persons per hectare, 42% spoke a language other than English at home and 16% had a low household income [35].

The current study utilised baseline data from self-reported cross-sectional resident surveys completed by adults in April–May 2013 living within 5 km or with children attending (pre) school within 3 km of these two parks. The REVAMP survey has been described in more detail elsewhere [33]. Respondents with at least one child aged 2–15 years living in the household, were also asked to complete proxy-report survey questions regarding their child’s use of parks and related behaviour.

Recruitment was via schools and postal survey. Pre-schools, primary and secondary government and Catholic schools located within 3 km of each park were invited to participate. Six pre-schools, ten primary schools and two secondary schools were recruited, which equated to approximately 5000 families (2500 from each park area) with children aged 2–15 years. Once schools consented to participate, a survey was sent home to: all families at each preschool with a child aged 2 years or older; every family at each primary school; and families at each secondary school with a student in school years 7–9. In addition, a random selection of 5000 residents (*n* = 2500 from each park area) who lived within a 5 km buffer of the two parks were identified by the two City Councils within which the parks were located and were mailed a survey.

In total, 9694 surveys were delivered, 37 were returned to sender, and 1487 surveys were returned completed (15.4% response rate; 15.1% intervention park, 15.7% control park), with 866 surveys including data on children. Response rates from schools and postal surveys were similar [33]. Survey items are presented in Additional file 1.

### Socio-demographic variables

The survey assessed parental age, sex, highest level of education, marital status, employment status, country of birth, parental status and dog ownership as well as child’s age, sex and extent of independence when walking to nearby parks (see Tables 1 and 2).

**Table 1:** Adult participants’ characteristics

	*N* = 1085
Gender, n (%)	
Male	316 (29.1%)
Female	769 (70.9%)
Age (years) - mean [SD]	48.9 [13.4]
Residential location, n (%)	
Lives within 5 km of intervention park	505 (46.5%)
Lives within 5 km of control park	580 (53.5%)
Education level, n (%)	
No formal qualifications	106 (9.8%)
Year 12/apprentice/diploma	317 (29.2%)
University/higher	662 (61.0%)
Employment status, n (%)	
Full-time work	366 (33.7%)
Part-time work or study	333 (30.8%)
Not working	386 (35.6%)
Marital status, n (%)	
Married/de facto	891 (82.3%)
Separated/widowed/divorced	138 (12.7%)
Never married	54 (5.0%)
Country of birth, n (%)	
Australia	729 (67.3%)
Other	354 (32.7%)
Dog ownership, n (%)	391 (36.0%)
Child under 2 years, n (%)	97 (8.9%)
Child between 2 and 15 years, n (%)	670 (61.8%)
Satisfaction with parks, n (%)	
Strongly disagree	38 (3.5%)
Disagree	152 (14.0%)
Neither agree nor disagree	112 (10.3%)
Agree	550 (50.7%)
Strongly agree	233 (21.5%)
Walking distance to park, n (%)	
1–5 min	514 (47.4%)
6–10 min	326 (30.0%)
11–20 min	171 (15.8%)
21–30 min	45 (4.1%)
31+ mins	29 (2.7%)
Visited park in past 7 days, n (%)	691 (63.7%)
Minutes/week LTPA – mean [SD], median [IQR]	217.2 [298.3], 120.0 [0.0–300.0]
Minutes/week TPA & LTPA (*n* = 833) – mean [SD], median [IQR]	348.7 [412.5], 215.0 [90.0–450.0]

**Table 2:** Child participants’ characteristics

	*N* = 753
Child’s sex, n (%)	
Male	387 (51.4)
Female	366 (48.6)
Child’s age (years) – mean [SD]	8.8 [3.7]
Residential location, n (%)	
Lives within 5 km of intervention park	332 (44.1)
Lives within 5 km of control park	421 (55.9)
Adult education level, n (%)	
No formal qualifications	47 (6.2)
Year12/apprentice/diploma	219 (29.1)
University/higher	487 (64.7)
Adult employment status, n (%)	
Full-time work	246 (32.7)
Part-time work or study	284 (37.7)
Not working	223 (29.6)
Marital status, n (%)	
Married/de facto	664 (88.4)
Separated/widowed/divorced	66 (8.8)
Never married	21 (2.8)
Country of birth, n (%)	
Australia	539 (71.7)
Other	213 (28.3)
Dog ownership, n (%)	277 (36.8)
Other child in household, n (%)	597 (79.3)
Child visits park without adult (i.e. alone or with friends/siblings), n (%)	271 (36.0)
Satisfaction with parks for child, n (%)	
Strongly disagree	24 (3.2)
Disagree	69 (9.2)
Neither agree nor disagree	90 (12.0)
Agree	391 (51.9)
Strongly agree	179 (23.8)
Child visited park in past 7 days, n (%)	496 (65.9)
Parks within walking distance for child, n (%)	712 (94.6)
Time child spent outside (minutes/week) - mean [SD], median [IQR]	652.8 [462.9],
	540.0 [300.0–900.0]
Days child met physical activity recommendations (5-15 yrs., *n* = 591) – mean [SD], median [IQR]	4.2 [2.0], 4.0 [3.0–6.0]

### Outcome variables

For adults, the primary outcome variable was self-reported LTPA in the past 7 days, and the secondary outcome combined TPA and LTPA. For children, the primary outcome variable was time spent outdoors in the past 7 days and the secondary outcome was the number of days in this period on which physical activity recommendations were met.

For adults, LTPA and TPA were assessed using the long version of the International Physical Activity Questionnaire, which has acceptable reliability and validity [36]. Total duration (minutes) per week of LTPA was computed by summing time spent walking (not including walking for transport) and in moderate- and vigorous-intensity physical activity in the past **7** days. Total duration per week of TPA was computed by summing time spent walking or cycling to travel from place to place in the past **7** days. TPA and LTPA were summed to create total time spent in TPA and LTPA.

Parents were asked “During the last 7 days, how many hours/minutes in total did your child spend outside (Monday to Friday, excluding time spent at school, pre-school or child care settings). The same question was asked for weekend days. Outdoor time has been consistently shown to be positively related to physical activity among children [37]. Participants who had a child aged 5–15 years were also asked “Over the past 7 days, on how many days did your 5–15 year old child participate in sport, physical activity or active play for at least 60 min per day” [38].

### Park satisfaction (exposure variable)

Participants were asked to report agreement with the following statement: “I am satisfied with the overall quality of the parks in my neighbourhood”. Response options were: 1 = strongly disagree to 5 = strongly agree. Test-re-test reliability for this item has been previously reported [33]. Participants also reported their satisfaction with the overall quality of available parks in their neighbourhood for their child. Neighbourhood was defined as everywhere within a 10–15 min walk from home.

### Park availability (exposure variable)

Participants were asked: “About how long would it take you to walk from home to the nearest park?” Response options adapted from the Neighborhood Environment Walkability Scale (NEWS) which has been shown to have acceptable reliability [39] were: 1) 1–5 min; 2) 6–10 min; 3) 11–20 min; 4) 21–30 min; 5) 31+ minutes. Responses were reversed scored, with higher scores representing a shorter distance to the park. Participants also reported whether there were any parks/playgrounds within walking distance from home for their child (yes/no).

### Park visitation (potential mediator)

Participants reported whether they had visited any park in the past 7 days (yes/no). Participants also answered the same question in relation to their child’s park visitation.

### Data analysis

For analysis using the primary outcome (adults = LTPA, children = time outdoors), only participants with complete data for the primary outcome, exposure variables, mediators and potential confounders were included (adults *n* = 1085, children *n* = 753). For the secondary outcome (adults = combined TPA and LTPA, children = days met physical activity recommendations), only participants with complete data for the secondary outcome, exposure variables, mediators and potential confounders were included (adults *n* = 833, children *n* = 591).

Analyses were conducted in Stata 15 (StataCorp, 2017, College Station, Texas) separately for adults and children. Analyses examined: 1) associations between (each of) park availability and park satisfaction and the outcome variables (*c-path*/total effect); 2) associations between (each of) park availability and park satisfaction and the potential mediator (*a-path*); 3) associations between the potential mediator and the outcome variable, adjusting for park availability and park satisfaction (*b-path*); and 4) the direct effect of (each of) park availability and park satisfaction on the outcome variable, adjusting for the potential mediator. The indirect mediated effect is the product of *a and b-path* coefficients (*a × b*) and provides an estimate of the relative strength of the mediation effect.

All models were adjusted for potential confounders. For adults, potential confounders were age, sex, education level, employment status, dog ownership, residential location (i.e. living near the intervention or control park) and whether they had children aged under 2 years and between 2 and 15 years. For children, potential confounders included age and sex of child, occupation and education level of responding adult, dog ownership, residential location, extent of independence when walking to nearby parks, and presence of other children in the household < 15 years. Models that examined park satisfaction as the exposure variable also included park availability as a potential confounder.

As the models contained continuous exposure and outcome variables and a binary mediator, the binary_mediators package in Stata was used to perform the analysis. This package applies a method for estimating the indirect effect as recommended by Mackinnon and Dwyer [40]. Under the application of this method, where the response variable for a model is continuous, an OLS regression is conducted. If the variable is binary, a logistic regression model is used. The package produces estimates of the total, direct, and indirect effects of the exposure on the outcome, presented as standardised regression coefficients. Estimates of the standard errors for the effects, and the corresponding percentile-based 95% confidence intervals were obtained using the bootstrap procedure, with 2000 replications specified. Although inferences are based on results from the mediation models, to assist with the interpretation of results, results from models fitted to estimate the *a and b-paths* will be presented as unstandardized coefficients.

The outcome variables for adults had skewed distributions, with high percentages of zeros; however, the sample was deemed sufficiently large for large-sample properties to hold (i.e., linear regression will produce valid results even for non-normal outcome data) [41]. Nonetheless, sensitivity analyses were conducted in which the sample was reduced to only include participants who indicated they performed some physical activity (i.e. > 0 mins) during the past 7 days and the outcome variables (LTPA, or combined TPA and LTPA) were then log-transformed for analysis.

## Results

Demographic characteristics are presented in Table 1 (adults) and Table 2 (children). The mean age of adults was 48.9 (SD 13.4) years and 70.9% were female. The mean age of the children was 8.8 (SD 3.7) years and 48.6% were female. More than 75% of adults lived within a 10 min walk of a park and 95% of children were within walking distance of a park from home. Participants viewed their neighbourhood parks favourably and visited often; 72% of adults reported being satisfied with the overall quality of parks in their neighbourhood and 64% reported visiting a park in the last 7 days. The results were slightly higher for children (76% satisfied and 66% visited in last 7 days).

### Adults

As shown in Table 3, there were significant positive effects of park availability on park visitation (*a-path*). There were also significant positive effects of park satisfaction on park visitation (*a-path*). The effects of visiting a park on both LTPA and combined TPA and LTPA, adjusting for either park availability or satisfaction, were also positive (*b-path*).

**Table 3:** Park visitation as a mediator of associations of park availability and park satisfaction with physical activity/time outdoors among children and adults

	Association between exposure and potential mediator^a^ *(a-path)* OR (95% CI)	Association between potential mediator^a^ and outcome^b^ variables *(b-path)* (95%CI)	Associations between exposure and outcome^b^ variables *(c-path)* (95%CI) Total effect*	Direct effect of exposure on outcome^b^ variables *(c’-path)* (95%CI) Direct effect*	Indirect effect of exposure on outcome^b^ variables *(a* × *b)* (95%CI) Indirect effect*
**Park availability (exposure variable 1)**					
Adult: LTPA^b^	**1.36 (1.19,1.55)**	**102.02 (63.90, 140.15)**	−0.026 (−0.094, 0.040)	−0.053 (−0.121, 0.010)	**0.028 (0.014, 0.045)**
Adult: TPA & LTPA^b^	**1.37 (1.19, 1.59)**	**155.77 (95.73, 215.81)**	−0.045 (− 0.124, 0.027)	−0.077 (− 0.153, − 0.009)	**0.032 (0.016, 0.052)**
Child: Time outdoors^b^	**3.15 (1.52, 6.52)**	**119.10 (44.45, 193.75)**	0.000 (−0.074, 0.072)	− 0.017 (− 0.091, 0.053)	**0.017 (0.005, 0.037)**
Child: # Days ≥60mins PA^b^	**3.14 (1.52, 6.52)**	**0.70 (0.35, 1.05)**	0.041 (−0.033, 0.119)	0.016 (−0.056, 0.092)	**0.025 (0.006, 0.054)**
**Park satisfaction (exposure variable 2)**					
Adult: LTPA^b^	**1.35 (1.19, 1.54)**	**101.48 (62.97, 140.00)**	0.035 (−0.030, 0.101)	0.006 (−0.059, 0.072)	**0.029 (0.014, 0.046)**
Adult: TPA & LTPA^b^	**1.24 (1.07, 1.44)**	**158.32 (97.99, 218.66)**	−0.008 (− 0.089, 0.069)	−0.031 (− 0.111, 0.046)	**0.023 (0.007, 0.045)**
Child: Time outdoors^b^	1.18 (0.99, 1.41)	**120.65 (45.80, 195.50)**	−0.012 (− 0.091, 0.064)	−0.024 (− 0.103, 0.054)	**0.011 (0.001, 0.026)**
Child: # Days ≥60mins PA^b^	1.18 (0.99, 1.41)	**0.68 (0.33, 1.03)**	0.066 (−0.022, 0.161)	0.045 (−0.045, 0.136)	**0.021 (0.004, 0.043)**

95% CI = 95% confidence intervals. **BOLD** = significant associations

^a^ Potential mediator: park visitation in past 7 days. Exposure variables: park availability and park satisfaction

Binary logisitic regression models were used to assess *a-paths* and odds ratios (OR) are reported

^b^ Primary outcome variables (adult = LTPA, child = time spent outdoors); secondary outcome variables (adult = TPA and LTPA, child = # days met physical activity recommendations)

Adult models were adjusted for age, gender, education level, employment status, location of respondent (Intervention or Control) and whether there were children in the household aged under 2 years and between 2 and 15 years

Child models were adjusted for age of child, gender of child, occupation of responding adult, education level of responding adult, dog ownership, location of respondent, extent of independence (never/rarely without parent or other adult vs. sometimes without parent or other adult or more frequently), and whether there were other children in the household up to the age of 15 years

* Standardised effects and bootstrapped percentile-based 95% confidence intervals calculated with Stata’s binary_mediators package are shown

After adjusting for potential confounders, there was no evidence of a direct relationship between park availability and LTPA or combined TPA and LTPA (*c’-path*). There was also no evidence of a direct relationship between reported park satisfaction and LTPA or combined TPA and LTPA (*c’-path*). There were indirect (mediated) effects of park availability (standardized coefficient (std. coeff) = 0.028, 95% CI: 0.014, 0.045) and reported park satisfaction (std. coeff = 0.029, 95% CI: 0.014, 0.046) on LTPA through park visitation (*a x b*). There was also evidence that the relationships of park availability (std. coeff = 0.032 95% CI: 0.016, 0.052) and reported park satisfaction (std. coeff = 0.023, 95% CI: 0.007, 0.045) with combined TPA and LTPA were mediated through park visitation. The sensitivity analyses showed similar effects (data not shown).

### Children

As shown in Table 3, there was a significant positive effect of park availability on park visitation (*a-path*); however, the association between park satisfaction and park visitation was not significant. The effects of visiting a park on both time spent outdoors and the number of days meeting physical activity recommendations, while adjusting for either park availability or satisfaction, were also positive (*b-path*).

After adjusting for potential confounders, there was no evidence of a direct relationship between parent-reported park availability or park satisfaction on time spent outdoors by children, or the number of days children met physical activity recommendations (*c’-path*). There were indirect effects of park availability (std. coeff = 0.017, 95% CI: 0.005, 0.037) and park satisfaction (std. coeff = 0.011, 95% CI: 0.001, 0.026) on time spent outdoors through park visitation (*a x b*). There was also some indication that the relationship between park availability (std. coeff = 0.025 95% CI: 0.006, 0.054) and park satisfaction (std. coeff = 0.021, 95% CI: 0.004, 0.043) and the number of days meeting physical activity recommendations was mediated by park visitation.

## Discussion

This study addresses important research gaps as, to our knowledge, it is one of the first studies to examine whether relationships between reported park availability or park satisfaction and physical activity among adults and time spent outdoors among children were mediated by park visitation. Although there was no evidence of a relationship between perceptions of park availability or satisfaction and adults’ physical activity and children’s time spent outdoors, the results suggest that park visitation in the past 7 days was an important mediator for the relationship between park satisfaction and park availability and physical activity/time outdoors for both adults and children.

Consistent with previous research, adults who reported having parks closer to home and children who had a park within perceived walking distance from home were more likely to have visited a park in the past 7 days [10, 42]. However, associations between park satisfaction and park visitation were only significant among adults. Given that park satisfaction may be related to park quality in terms of amenities, maintenance and aesthetics [24] this finding is contrary to those from previous studies among adults and adolescents that suggest quality of parks is more important than quantity or availability for encouraging park use [27, 30, 43]. Inconsistencies amongst children’s park satisfaction may also be explained by subtle differences in park use and park feature requirement at different ages throughout childhood [26]. Future studies are required to better understand the qualities and features of parks that contribute to overall park satisfaction for different user groups.

Neither park availability nor park satisfaction had a significant impact on physical activity levels for adults or time spent outdoors/physical activity among children. Some previous studies have also concluded that there is little evidence to support a relationship between access to parks in the local area and overall physical activity [19, 44]. This suggests that availability of parks alone may not be sufficient to impact overall physical activity levels. It is essential that features that encourage active use for all demographic groups are understood and prioritised in the design of new parks and the refurbishment of existing parks [45, 46].

Although some natural experiment studies have shown increases in park-based physical activity after physical improvements to the park [47–49], a review of interventions to encourage physical activity in urban green space found there was more promising evidence to support physical changes to the built environment combined with programs for increasing green space use and park-based physical activity than changes to the built environment alone [50]. Therefore, additional individual- and community-level incentives may also be needed to motivate people to be active in parks. For example, governments and health professionals should consider encouraging park-based physical activity through community programs and tailored marketing [51].

This study had a cross-sectional design therefore causality cannot be inferred. It is possible that there are bi-directional associations, for example, frequency of visiting a park may affect park satisfaction, or participation in LTPA may influence park visitation if people visit the park to be active. Our model included only a binary measure of park visitation in the week prior to the survey, and this may not be indicative of habitual behaviour. For adults, the measure of park availability was based on self-reported distance to the nearest park and it is possible that the nearest park is not the one visited most often. Similarly, for children, the measure of park availability was based on the reported presence of parks/playgrounds within walking distance from home and this may not be the park they usually visit. The measure of park satisfaction was based on self-reported satisfaction with the overall quality of the parks in their neighbourhood for themselves and their child. Objective audits of park features and amenities to determine park quality may be a valuable component of future park research. Physical activity for adults was self-reported, although a reliable and valid measure was used [36], and proxy-reported for children; future studies may benefit from objective measures of physical activity and park availability. Future studies may also benefit from measuring park-based physical activity which is more context-specific than overall physical activity. Future research could also explore whether child’s age and gender moderate the associations examined.

## Conclusion

Given the potentially important role of parks in providing opportunities for physical activity, the findings suggest it may be important to improve park availability and user’s satisfaction with park quality in order to increase park visitation and consequently increase physical activity and time spent outdoors among adults and children. This is an important practical consideration for planners and managers particularly for parks that serve communities with high rates of families with children. Future studies with park users and non-park users of varying demographic characteristics living in urban and rural locations are required to better understand what features should be prioritised in park design to enhance park satisfaction.

## Supplementary Information

**Additional file 1.** Survey questions.

## Data Availability

The datasets used and/or analysed during the current study are available from the corresponding author on reasonable request.

## Abbreviations

CBD: Central business district
CI: Confidence interval
LTPA: Leisure-time physical activity
OR: Odds ratio
REVAMP: Recording and EValuating Activity in a Modified Park
SD: Standard deviation
Std. coeff: Standardized coefficient
TPA: Transport-related physical activity

## References

[1] Sallis JF, Cerin E, Conway TL, Adams MA, Frank LD, Pratt M (2016). Physical activity in relation to urban environments in 14 cities worldwide: a cross-sectional study. Lancet..

[2] van den Bosch M, Ode SA (2017). Urban natural environments as nature-based solutions for improved public health - a systematic review of reviews. Environ Res.

[3] Koohsari MJ, Mavoa S, Villanueva K, Sugiyama T, Badland H, Kaczynski AT (2015). Public open space, physical activity, urban design and public health: concepts, methods and research agenda. Health Place..

[4] Evenson KR, Fang WEN, Hillier AMY, Cohen DA (2013). Assessing the contribution of parks to physical activity using global positioning system and Accelerometry. Med Sci Sports Exerc.

[5] Veitch J, Carver A, Abbott G, Giles-Corti B, Timperio A, Salmon J (2015). How active are people in metropolitan parks? An observational study of park visitation in Australia. BMC Public Health.

[6] Joseph RP, Maddock JE (2016). Observational Park-based physical activity studies: a systematic review of the literature. Prev Med.

[7] Oliveira AF, Moreira C, Abreu S, Mota J, Santos R (2014). Environmental determinants of physical activity in children: a systematic review. Arch Exerc Health Dis.

[8] Limstrand T (2008). Environmental characteristics relevant to young people's use of sports facilities: a review. Scand J Med Sci Sports.

[9] Gardsjord H, Tveit M, Nordh H (2014). Promoting youth’s physical activity through park design: linking theory and practice in a public health perspective. Landsc Res.

[10] Ding D, Sallis JF, Kerr J, Lee S, Rosenberg DE (2011). Neighborhood environment and physical activity among youth a review. Am J Prev Med.

[11] Kaczynski AT, Henderson KA (2007). Environmental correlates of physical activity: a review of evidence about parks and recreation. Leis Sci.

[12] Durand CP, Andalib M, Dunton GF, Wolch J, Pentz MA (2011). A systematic review of built environment factors related to physical activity and obesity risk: implications for smart growth urban planning. Obes Rev.

[13] Owen N, Humpel N, Leslie E, Bauman A, Sallis JF (2004). Understanding environmental influences on walking; review and research agenda. Am J Prev Med.

[14] Veitch J, Abbott G, Kaczynski AT, Wilhelm Stanis SA, Besenyi GM, Lamb KE (2016). Park availability and physical activity, TV time, and overweight and obesity among women: findings from Australia and the United States. Health Place.

[15] Kaczynski AT, Besenyi GM, Wilhelm Stanis SA, Koohsari MJ, Oestman KB, Bergstrom R (2014). Are park proximity and park features related to park use and park-based physical activity among adults? Variations by multiple socio-demographic characteristics. Int J Behav Nutr Phys Act.

[16] Witten K, Hiscock R, Pearce J, Blakely T (2008). Neighbourhood access to open spaces and the physical activity of residents: a national study. Prev Med.

[17] Koohsari MJ, Kaczynski AT, Giles-Corti B, Karakiewicz JA (2013). Effects of access to public open spaces on walking: is proximity enough?. Landscape Urban Plann..

[18] Bancroft C, Joshi S, Rundle A, Hutson M, Chong C, Weiss CC (2015). Association of proximity and density of parks and objectively measured physical activity in the United States: a systematic review. Soc Sci Med.

[19] Flowers EP, Freeman P, Gladwell VF (2016). A cross-sectional study examining predictors of visit frequency to local green space and the impact this has on physical activity levels. BMC Public Health.

[20] Marquet O, Hipp JA, Alberico C, Huang JH, Fry D, Mazak E (2019). Short-term associations between objective crime, park-use, and park-based physical activity in low-income neighborhoods. Prev Med..

[21] Han B, Cohen DA, Derose KP, Li J, Williamson S (2018). Violent crime and park use in low-income urban neighborhoods. Am J Prev Med.

[22] Bedimo-Rung AL, Mowen AJ, Cohen DA (2005). The significance of parks to physical activity and public health: a conceptual model. Am J Prev Med.

[23] McCormack GR, Rock M, Toohey AM, Hignell D (2010). Characteristics of urban parks associated with park use and physical activity: a review of qualitative research. Health Place..

[24] Bai H, Wilhelm Stanis SA, Kaczynski AT, Besenyi GM (2013). Perceptions of neighborhood park quality: associations with physical activity and body mass index. Ann Behav Med.

[25] Veitch J, Salmon J, Ball K (2008). Children's active free play in local neighborhoods: a behavioral mapping study. Health Educ Res.

[26] Flowers EP, Timperio A, Hesketh KD, Veitch J (2019). Examining the Features of Parks That Children Visit During Three Stages of Childhood. Int J Env Res Public Health..

[27] Giles-Corti B, Broomhall M, Knuiman M, Collins C, Douglas K, Ng K (2005). Increased walking. How important is distance to, attractiveness, and size of public open space?. Am J Prev Med.

[28] Sugiyama T, Francis J, Middleton NJ, Owen N, Giles-Corti B (2010). Associations between recreational walking and attractiveness, size, and proximity of neighborhood open spaces. Am J Public Health.

[29] Van Cauwenberg J, Cerin E, Timperio A, Salmon J, Deforche B, Veitch J (2015). Park proximity, quality and recreational physical activity among mid-older aged adults: moderating effects of individual factors and area of residence. Int J Behav Nutr Phys Act.

[30] Ries AV, Voorhees CC, Roche KM, Gittelsohn J, Yan AF, Astone NM (2009). A quantitative examination of park characteristics related to park use and physical activity among urban youth. J Adolesc Health.

[31] Huang JH, Hipp JA, Marquet O, Alberico C, Fry D, Mazak E (2020). Neighborhood characteristics associated with park use and park-based physical activity among children in low-income diverse neighborhoods in New York City. Prev Med..

[32] Veitch J, Ball K, Crawford D, Abbott G, Salmon J (2013). Is park visitation associated with leisure-time and transportation physical activity?. Prev Med.

[33] Veitch J, Salmon J, Carver A, Timperio A, Crawford D, Fletcher E (2014). A natural experiment to examine the impact of park renewal on park-use and park-based physical activity in a disadvantaged neighbourhood: the REVAMP study methods. BMC Public Health.

[34] .id the population experts. Brimbank City Council, community profile. Available from: https://profile.id.com.au/brimbank. Accessed 1 Feb 2021.

[35] .id the population experts. Manningham City Council, community profile. Available from: https://profile.id.com.au/manningham. Accessed 1 Feb 2021.

[36] Craig CL, Marshall AL, Sjostrom M, Bauman AE, Booth ML, Ainsworth BE (2003). International physical activity questionnaire: 12-country reliability and validity. Med Sci Sports Exerc.

[37] Raza W, Forsberg B, Johansson C, Sommar JN (2018). Air pollution as a risk factor in health impact assessments of a travel mode shift towards cycling. Glob Health Action.

[38] Australian Government Department of Health. Australia's Physical Activity and Sedentary Behaviour Guidelines and the Australian 24-Hour Movement Guidelines 2019. Available from: https://www1.health.gov.au/internet/main/publishing.nsf/Content/health-pubhlth-strateg-phys-act-guidelines. Accessed 1 Feb 2021.

[39] Cerin E, Sit CHP, Barnett A, Huang WYJ, Gao GY, Wong SHS (2017). Reliability of self-report measures of correlates of obesity-related behaviours in Hong Kong adolescents for the iHealt(H) and IPEN adolescent studies. Arch Public Health.

[40] Mackinnon DP, Dwyer JH (1993). Estimating mediated effects in prevention studies. Eval Rev.

[41] Lumley T, Diehr P, Emerson S, Chen L (2002). The importance of the normality assumption in large public health data sets. Annu Rev Public Health.

[42] Calogiuri G, Chroni S (2014). The impact of the natural environment on the promotion of active living: an integrative systematic review. BMC Public Health.

[43] Sugiyama T, Gunn LD, Christian H, Francis J, Foster S, Hooper P (2015). Quality of public open spaces and recreational walking. Am J Public Health.

[44] Ord K, Mitchell R, Pearce J (2013). Is level of neighbourhood green space associated with physical activity in green space?. Int J Behav Nutr Phys Act.

[45] Sugiyama T, Carver A, Koohsari MJ, Veitch J (2018). Advantages of public green spaces in enhancing population health. Landscape Urban Plann.

[46] Costigan SA, Veitch J, Crawford D, Carver A, Timperio A (2017). A Cross-Sectional Investigation of the Importance of Park Features for Promoting Regular Physical Activity in Parks. Int J Environ Res Public Health..

[47] Veitch J, Ball K, Crawford D, Abbott GR, Salmon J (2012). Park improvements and park activity: a natural experiment. Am J Prev Med.

[48] Veitch J, Salmon J, Crawford D, Abbott G, Giles-Corti B, Carver A (2018). The REVAMP natural experiment study: the impact of a play-scape installation on park visitation and park-based physical activity. Int J Behav Nutr Phy.

[49] Cohen DA, Han B, Isacoff J, Shulaker B, Williamson S (2019). Renovations of neighbourhood parks: long-term outcomes on physical activity. J Epidemiol Community Health.

[50] Hunter RF, Christian H, Veitch J, Astell-Burt T, Hipp JA, Schipperijn J (2015). The impact of interventions to promote physical activity in urban green space: a systematic review and recommendations for future research. Soc Sci Med.

[51] Groshong L, Stanis SA, Kaczynski AT, Hipp JA, Besenyi GM (2017). Exploring attitudes, perceived norms, and personal agency: insights into theory-based messages to encourage park-based physical activity in low-income urban neighborhoods. J Phys Act Health.

