# Supplementary material for: Are park availability and satisfaction with neighbourhood parks associated with physical activity and time spent outdoors?
Source: BMC Public Health. 2021 Feb 6;21:306. doi: 10.1186/s12889-021-10339-1 (PMC7866776; doi:10.1186/s12889-021-10339-1)
Supplement: Supplementary file 1 — Additional file 1. Survey questions. [file 12889_2021_10339_MOESM1_ESM.pdf]

Additional File 1: Survey questions

This section of the survey relates to your local neighbourhood environment. By neighborhood we mean everywhere within a 10-15 minute walk from your home. When answering the questions please make sure you refer to your neighbourhood only.

The following statements are about parks in your neighbourhood. By park, we mean a public park or outdoor recreation area that is designed for active or passive use. How much do you agree or disagree with the following statements? (Please shade one response on each line)

		Strongly disagree	Disagree	Neither agree or	Agree	Strongly agree	Don't know	N/A
A	I am satisfied with the overall quality of parks in my neighbourhood							

Have you visited a park in the past 7 days?

Yes	
No	

About how long would it take to walk from home to the following places?

(Please shade one response on each line)

		1-5 mins	6-10 mins	11-20 mins	21-30 mins	31+ mins	Don't know	N/A
A	Nearest park to home							
B	Park you visited most often in the past 3 months (not including Brimbank Park)							
C	Brimbank Park							

These questions are about how you travelled from place to place, including to places like work, shops, movies and so on.

During the last 7 days, on how many days did you bicycle for at least 10 minutes at a time to go from place to place? (Write 0 if you did not do this activity)

☐ days per week

How much time did you usually spend on ONE OF THOSE DAYS to bicycle from place to place?

☐☐ hours and ☐☐ minutes/day

During the last 7 days, on how many days did you walk for at least 10 minutes at a time to go from place to place? (Write 0 if you did not do this activity)

☐ days per week

How much time did you usually spend on ONE OF THOSE DAYS walking from place to place?

☐☐ hours and ☐☐ minutes/day

This section is about all the physical activities that you did in the last 7 days solely for recreation, sport, exercise or leisure. Please do not include any activities you have already mentioned.

Not counting any walking you have already mentioned, during the last 7 days, on how many days did you walk for at least 10 minutes at a time in your leisure time? (Write 0 if you did not do this activity)

☐ days per week

How much time did you usually spend on ONE OF THOSE DAYS walking in your leisure time?

☐☐ hours and ☐☐ minutes/day

Think about only those physical activities that you did for at least 10 minutes at a time. During the last 7 days, on how many days did you do vigorous physical activities like aerobics, running, fast bicycling, or fast swimming in your leisure time? (Write 0 if you did not do this activity)

☐ days per week

How much time did you usually spend on ONE OF THOSE DAYS doing vigorous physical activities in your leisure time?

☐☐ hours and ☐☐ minutes/day

Think about only those physical activities that you did for at least 10 minutes at a time. During the last 7 days, on how many days did you do moderate physical activities like bicycling at a regular pace, swimming at a regular pace or doubles tennis in your leisure time? (Write 0 if you did not do this activity)

☐ days per week

How much time did you usually spend on ONE OF THOSE DAYS doing moderate physical activities in your leisure time?

☐☐ hours and ☐☐ minutes/day

What is your sex?

**Male**	
**Female**	

What is your date of birth? (dd/mm/yyyy)

Which of the following best describes your current relationship status? (Please shade one response only)

A	In a registered marriage	
B	In a de facto relationship	
C	Separated	
D	Divorced	
E	Widowed	
F	Never married	

Which of the following best describes your current MAIN DAILY activities and/or responsibilities, and those of your spouse/partner if you have one? (Please shade one response in each column: one for you, and one for your spouse/partner. If you do not have a spouse/partner please shade that response below)

		**Self**	**Spouse/**
A	Working full-time		
B	Working part-time		
C	Unemployed		
D	Keeping house and/or raising children full-time		
E	Studying		
F	Retired		
G	I do not have a spouse or partner		

Where were you born?

A	Australia	
B	China	
C	Greece	
D	India	
E	Italy	
F	Malta	

G	UK or Ireland	
H	Vietnam	
I	Other (please specify):	

Do you own a dog?

Yes	
No	

Do you have a child aged under 2 years living with you now?

Ye:		If yes, how many

Do you have any children aged between 2 and 15 years living with you now?

Yes	
No	

How many children aged 2-15 years live with you now? (Please shade all that apply)

		None	One	Two	Three or more
A	Between 2 and 4 years				
B	Between 5 and 15 years				

If you have more than one child aged between 2-15 years living with you now, please only answer the following questions in relation to the child who has the NEXT BIRTHDAY.

Child's sex:

Male	
Female	

Child's date of birth? (dd/m/yyyy)

What is your relationship to this child? (Please shade one response only)

A	Mother		
B	Father		
C	Grandparent		
D	Stepmother/father		
E	Guardian		
F	Other (please specify):		

This question is about your satisfaction with parks and playgrounds in your local neighbourhood IN RELATION TO YOUR CHILD. How much do you agree or disagree with the following statements?

(Please shade one response on each line)

		Strongly disagree	Disagree	Neither agree or disagree	Agree	Strongly agree	Don't know
C	I am satisfied with the overall quality of available parks **for my child** in my neighbourhood						

Are there any parks/playgrounds that are within walking distance from home for your child?

Yes	
No	

How often does your child usually walk to nearby parks/playgrounds with the following people?

(Please shade one response per line)

		Never	Rarely	Sometimes	Often	Very often
A	By him/herself					
B	With parent/other adult					
C	With friends/siblings					

Has your child visited a park in the past 7 days?

Yes	
No	

During the last 7 days, how many hours/minutes in TOTAL did your child spend outside?

(MONDAY to FRIDAY, excluding time spent at school, pre-school or child care settings)

☐☐ hours and ☐☐ minutes/week (Monday to Friday)

During the last 7 days, how many hours/minutes in TOTAL did your child spend OUTSIDE on the weekend? (SATURDAY and SUNDAY)

☐☐ hours and ☐☐ minutes/week (Saturday and Sunday)

If your child is aged 2-5 years and has not yet started school, please skip to Question x

Over the past 7 days, on how many days did your <u>5-15 year old child</u> participate in sport, physical activity, or active play for a total of <u>at least 60 minutes per day</u>? This may be done in sports, school activities, or walking to school and could include netball, basketball, swimming, dancing, tennis, riding a bike, or playing at the park. The 60 minutes does not need to be done all at once but is a total over the whole day e.g. 20 minutes in the morning, 20 minutes at school and 20 minutes in the afternoon.

0 days	1 day	2 days	3 days	4 days	5 days	6 days	7 days
